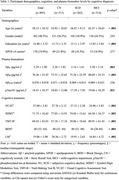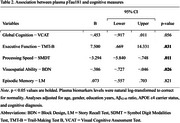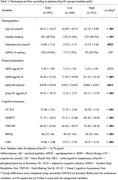# Association of Plasma *p*‐Tau181 and Cognition in Non‐Demented Individuals: Insights from the BIOCIS Study in Southeast Asia

**DOI:** 10.1002/alz70856_099201

**Published:** 2025-12-25

**Authors:** Timothy Aw Bang Hao, Yi Jin Leow, Nagaendran Kandiah

**Affiliations:** ^1^ Dementia Research Centre (Singapore), Lee Kong Chian School of Medicine, Nanyang Technological University, Singapore, Singapore; ^2^ National University of Singapore, Singapore, Singapore; ^3^ Neuroscience and Mental Health Programme, Lee Kong Chian School of Medicine, Nanyang Technological University, Singapore, Singapore; ^4^ National Healthcare Group, Singapore, Singapore

## Abstract

**Background:**

Tau pathology is a driving force behind cognitive decline in the early stages of Alzheimer's disease (AD). While amyloid‐β (Aβ) amplifies tau spread and neurotoxicity, tau accumulation may occur independently of and at subthreshold Aβ levels. Tau‐PET imaging and cerebrospinal fluid (CSF) biomarkers strongly link tau pathology and early cognitive decline. However, these methods are invasive, costly, and impractical for widespread use. Plasma phosphorylated tau 181 (pTau181) offers a promising alternative, with growing evidence supporting its ability to detect both tau and amyloid pathology. This study explored the relationship between plasma pTau181 levels and cognition in non‐demented individuals with subthreshold Aβ from a Southeast Asian cohort.

**Method:**

This study included 893 participants from the Biomarkers and Cognition Study, Singapore (BIOCIS), with a mean age of 58.1 years (SD = 10.5), 40.5% male, and a mean education level of 14.9 years (SD = 3.5). Of these, 317 were classified as cognitively normal (CN), 262 had subjective cognitive decline (SCD), and 214 had mild cognitive impairment (MCI). Only individuals with subthreshold Aβ (Aβ_42/40_ ratio ≥ 0.05) were included. Multiple regression analyses examined the relationship between plasma pTau181 and cognition, adjusting for age, gender, education years, Aβ_42/40_ ratio, APOE ε4 carrier status, and cognitive diagnosis.

**Result:**

Elevated plasma pTau181 levels were significantly associated with lower executive function (*B* = 7.50, 95% CI [0.67, 14.33], *p* = .0.31), processing speed (*B* = −3.29, 95% CI [−5.84, −.75], *p* = .011), and visuospatial ability (*B* = −.39, 95% CI [−.73, −.05], *p* = .026), suggesting an early vulnerability of cognition to tau accumulation. In contrast, plasma pTau181 was not significantly associated with global cognition (*B* = −.45, 95% CI [−.92, .01], *p* = .056) or episodic memory (*B* = .07, 95% CI [−.56, .70], *p* = .821), suggesting a domain‐specific effect.

**Conclusion:**

This study highlights that plasma pTau181 may serve as a minimally invasive biomarker for early cognitive deficits in executive function, processing speed, and visuospatial ability in non‐demented individuals with subthreshold amyloid pathology. Longitudinal research is needed to validate these findings and explore its predictive value for progression to AD and integration into early intervention strategies.